# Guiding catheter inner lumen damage during percutaneous coronary intervention

**DOI:** 10.1002/ccr3.4627

**Published:** 2021-08-16

**Authors:** Azriel Osherov, Jamal Jafari, Chaim Yosefy, Enrique Gallego‐Colon

**Affiliations:** ^1^ Interventional Cardiology Unit Barzilai Medical Center The Ben‐Gurion University of the Negev Ashkelon Israel; ^2^ Cardiology Department Barzilai University Medical Center Ben‐Gurion University Ashkelon Israel

**Keywords:** guide catheter, PCI complications, percutaneous coronary intervention, stenting technique

## Abstract

Guiding catheter damage and body wire intermingling are uncommon complications of standard operational procedures. Optimal application of this device includes replacing the small guiding catheter upon excessive resistance during stent insertion.

## Graphical abstract

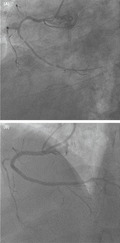

## 1 INTRODUCTION

Percutaneous coronary intervention (PCI) is increasingly employed for treatment of complex coronary artery disease. We present a rare case of stent entrapment within the 5F guiding catheter's inner lumen during PCI. This case report aims to help interventional cardiologists to recognize the associated risks of using small caliber‐guiding catheters.

Coronary artery intervention is based on using guiding catheters for cannulation, support, and safe delivery of stents over wires. Larger caliber‐guiding catheters (7F and 8F) increase bleeding complications, loss of radial pulse, and prevent “deep intubation” for stent delivery.^1^, ^2^ Small caliber‐guiding catheters (5F and 6F), however, are associated with increased friction and resistance during the passage of larger caliber stents or noncompliant balloons. In the present case, we describe a case of increased resistance during stent delivery when a small caliber‐guiding catheter is employed.

## 2 CASE REPORT

**FIGURE 1:**
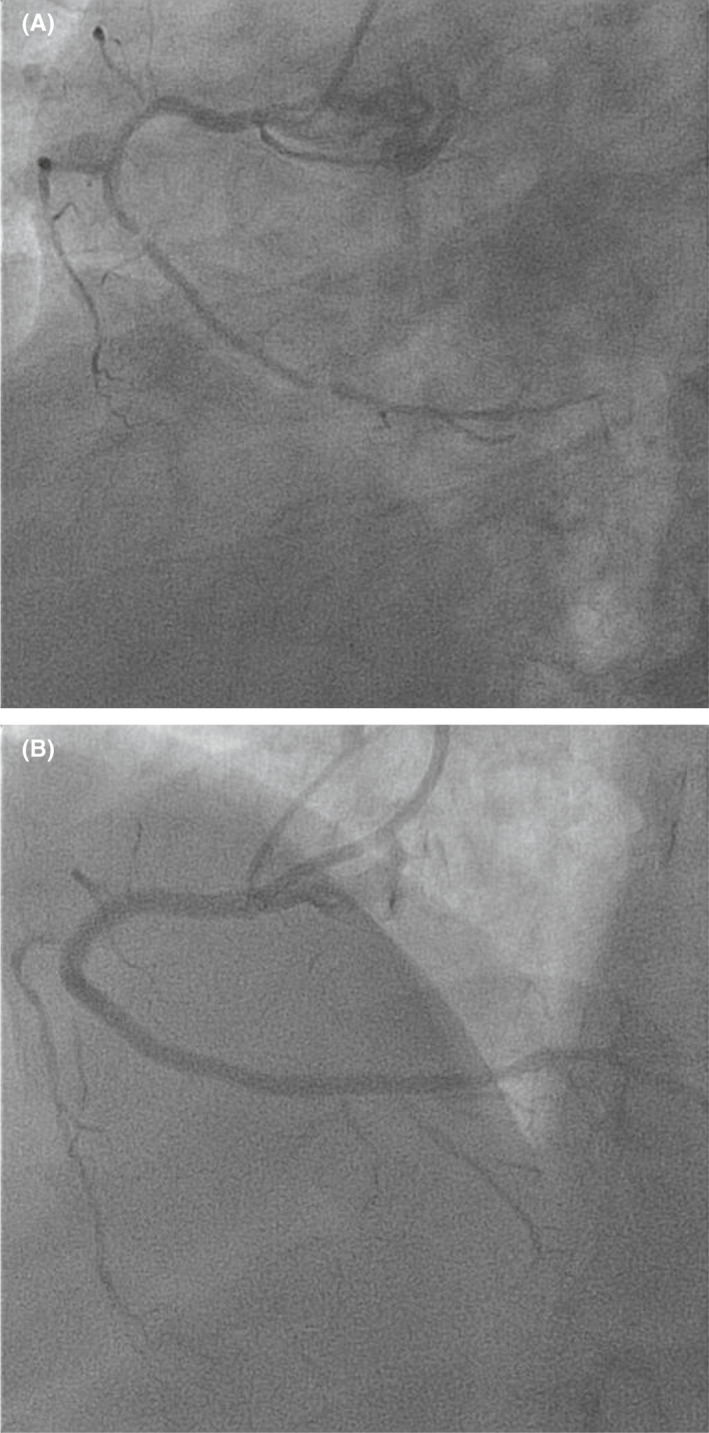
Selective coronary angiography of the right coronary artery before (A) and after (B) stent placement

**FIGURE 2:**
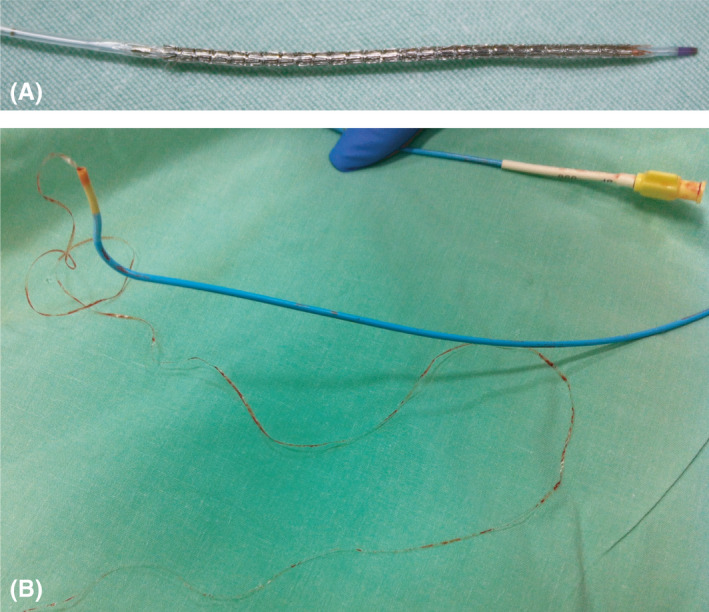
(A) Xience stent, 2.5 on 38 mm, after removal (Abbott Laboratories). (B) A 65 cm long nonopaque plastic sleeve attached to the distal part of the 5F AR2 guiding catheter (Cordis Corporation)

A 62‐years‐old man with history of heavy smoking, diabetes mellitus, hyperlipidemia, and obesity presented to the emergency department with chest discomfort and palpitations. The patient was admitted with unstable angina pectoris and paroxysmal atrial flutter. Coronary angiography was performed revealing three‐vessel coronary artery disease with 50%–70% stenosis of the proximal left anterior descending (LAD), 70%–80% stenosis of the distal part of the LAD, and 99% stenosis of the mid and distal right coronary artery (RCA) (Figure 1). Based on the clinical status, a decision was made to revascularize the RCA urgently, and the LAD in a second time a month later without any procedural complication. Standard trans‐radial access angioplasty of the RCA was initiated via a 5F Amplatz right 2 guide (AR2) (Cordis Corporation) with a 2.5 on 38 mm Xience stent (Abbott Laboratories). At first, the degree of stenosis prevented stent deployed. Despite the use of balloon angioplasty and body wire guiding, no advancement beyond the proximal part of the artery was achieved. No significant anatomical challenge, radial loop or subclavian tortuosity, was observed in this patient. During the second attempt to insert the stent through the guide, we encountered resistance at the proximal part of the guide. The potential damage of the struts was suspected and the stent removed. Upon visual inspection, the Xience stent was found intact except for minor flaring of struts on its proximal part (Figure 2A). Consequently, the 5F guide was replaced with a 6F Amplatz left 0.75 (AL 0.75) (Medtronic) guide. On removing the 5F guide, a long plastic sleeve of 1 mm wide and 65 cm long from the inner lumen of the guide was attached to the distal part of the 5F AR2 guiding catheter (Figure 2B). Angioplasty was resumed with the AL 0.75 guide, and a 4 Resolute Integrity drug eluting stent (Medtronic) was successfully deployed. No other complications such as embolization and/or myocardial injury were observed. The patient was later discharged with Prasugrel 10 mg (60 mg one‐time), Aspirin 100 mg, Metformin 850 mg, Atorvastatin 80 mg, and Ramipril 1.25 mg.

## 3 DISCUSSION

We present a rare case of stent entrapment within the 5F guiding catheter's inner lumen during PCI. Upon inspection, the inner lumen of the guiding catheter was peeled away during stent insertion causing body wire intermingling. Successful angioplasty and stent redeployment were achieved by replacing the 5F AR2 guide with a 6F AL 0.75. To our knowledge, this is the first report on inner lumen guiding catheter sheath being peeled apart allowing the wires to be released. The incident was reported to the United States Food and Drug Administration (FDA) and to the manufacturing company. The 5F AR2 guiding catheter was inspected by Cordis laboratories. The report indicated that an elongated part of the inner lumen was peeled off without any related manufacturing defects.

The damage of the inner lumen sheath is a seldom event; however, in this case report, several independent factors when combined may have contributed to the damage. First, significant resistance, as in our case, can cause the inner lumen to peel off. Second, the insertion of bulky and large stents or balloons through a narrowed guiding catheter can cause excessive friction within the inner lumen.^3^ In this case report, a large 38 mm stent may have contributed to the damage of the inner lumen. Another contributing factor is the inner diameter of the guiding catheter. To guarantee success, several options of steerable guide wires can be used for stent deployment in the occluded vessel. The 5F guiding catheters for radial access angioplasty and stenting procedures are commonly used during interventional cardiology due to reduced radial artery spasm and thrombosis.^4^ In our case, the internal diameter of the 5F guide catheter by Cordis was 0.056 inch (1.4 mm); nevertheless, the internal diameter of the 6F guide catheter was 0.071 inch (1.8 mm). All these factors associated with restrictive tortuosity of the subclavian artery, and the equipment inside the lumen including the body wire may have contributed to this case. Thus, upon excessive resistance we recommend removing and replacing the guiding catheter, since the inner lumen can be compromised. In addition, we consider that patients at risk for this complication are women with narrow radial arteries, or patients with a tortuous subclavian artery whom a small caliber guiding catheter could be selected for the procedure. The importance of replacing the guiding catheter is crucial, since the nonopaque plastic materials can emboli leading to coronary obstruction or cerebrovascular accidents. Our case report reveals that the risks involving standard guiding catheters should not be overlooked.

## 4 CONCLUSIONS

Small caliber guiding catheters have an important role in interventional cardiology. Unfortunately, the angioplasty guide‐wire and stent deployment, adjusted to the patient's clinical situation, are not without risks. Guiding catheter's inner lumen damage and body wire intermingling can occur during standard operating procedures. Thus, extra caution is important for optimal application of this device to avoid this seldom but potentially fatal event.

## CONFLICT OF INTEREST

None declared.

## AUTHOR CONTRIBUTIONS

AO and CY conceptualized and designed the study; AO and JJ involved in data acquisition; CY and EG‐C drafted the manuscript and revised it critically for important intellectual content. EG‐C and AO involved in analysis and interpretation. All authors approved the manuscript submitted.

## Data Availability

The data that support the findings of this study are available on request from the corresponding author. The data are not publicly available due to privacy or ethical restrictions.
